# Mental health and psychosocial support concerns among frontline workers within the Eastern and Southern Africa COVID-19 response

**DOI:** 10.11604/pamj.supp.2022.41.2.29032

**Published:** 2022-06-11

**Authors:** Ndeye Marie Diop, Ida Andersen, Brighton Gwezera, Jakob Julius Fihn, Fatima Gohar, Dorothy Morgos, Florence Baingana

**Affiliations:** 1 UNICEF East and Southern Africa Regional Office, Nairobi, Kenya,; 2 International Committee of the Red Cross, Geneva, Switzerland; 3 Regional Psychosocial Support Initiative, Randburg, South Africa; 4 World Health Organization, Brazzaville, Republic of Congo

**Keywords:** Healthcare worker, mental Health, Southern Africa, psychosocial support, COVID-19

## Abstract

We carried out a mental health assessment survey of frontline workers in Eastern and Southern Africa regarding COVID-19 pandemic in the region. A total of 723 people responded to the anonymous survey which was available in English, French and Portuguese. Two thirds of respondents felt overwhelmed and the remaining one third expressed fear of the pandemic. Concern about self and one´s wellbeing was associated with the feeling of being supported by one´s supervisor. Frontline workers that acknowledged supervisor support also expressed a significantly better wellbeing than others that did not receive supportive supervision. It is important to strengthen supervisors´ capacity for psychological support to their subordinates. It is also necessary to emphasise the importance of giving attention to staff mental health concerns. Supervisors should provide information on referral opportunities and encourage their staff to take advantage of them when in need of specialised services. While frontline workers have been celebrated worldwide for their efforts during the COVID-19 pandemic, reports also indicate that some of them are exposed to stigma, discrimination and even violence within their communities, at workplace and surroundings. Further studies will improve current understanding of the mental health and psychological concerns other categories of professional caregivers experienced while responding to the pandemic.

## Introduction

The speed with which COVID-19 spread across the globe, the number of cases and deaths reported, the media attention to it, and the strict containment measures that were announced, and the strategies employed to ensure compliance were a major source of stress and anxiety. The burden of the stress and concern is felt more by frontline workers who are in contact with affected individuals. Most at risk are the frontline workers providing health care to the infected, contact tracers and social workers, in close contact with the disease and those suffering form it [1]. Factors causing additional levels of stress and concern vary but include the rapid spread of the virus, the uncertainty surrounding the modes of transmission and the lack of a definitively proven and effective treatment [2].

## Methods

Between 18 May and 12 June 2020 we carried out a rapid survey to assess the impact of the COVID-19 pandemic on the mental wellbeing of front workers in Eastern and Southern Africa. The survey assessed the mental health concerns and needs of frontline workers and determined the capacity of frontline workers to provide Mental Health and Psychosocial Support services (MHPSS) to those affected by COVID-19 as well as the frontline workers. The survey focused on assessment of the mental health concerns of frontline workers, the readiness to support their peers and the capacity to provide for their clients. The survey was hosted on Microsoft Forms and disseminated through a mixed use of convenience and snowball sampling. The survey link was sent through WhatsApp platform to groups and contacts at regional and country offices in the East and Southern Africa Region (ESAR). The survey was anonymous, voluntary, and available in English, French and Portuguese. The survey questionnaire was sent to a wide range of professionals, including health and social workers who were frontline responders in the COVID-19 pandemic intervention at country level. The respondents were grouped into five overarching categories to ease the analysis of data. Logistic regression models were used for computing odds ratios (OR) with corresponding 95% Confidence Intervals (CIs) and p-values from the Wald test in order to measure associations between variables.

## Results

Of the 723 respondents, 49% were female and 51% were male. Health care workers such as medical doctors, midwives and nurses classified as clinicians were 35%. Psychiatrists, psychologists and counsellors classified as mental health professionals were 13%. Epidemiologists, public health employees who we classified broadly as public health professionals were 4%. Child protection officers, social and welfare workers were classified as social workers (36%) and 12% “others” included administrative and support staff.

### Emotional impact of COVID-19

Among the 723 respondents, 68% felt overwhelmed, stressed or anxious while 33% reported feeling scared. One in five respondents noted feelings of sadness, confusion, lack of control and anger. Respondents also reported experiencing headaches (24%), problems sleeping (20%), heart racing (7%) and dizziness (5%) during the COVID-19 Response. Women were more likely than males to report feeling sad (OR 1.85, p = 0.001), scared (OR 1.49, p = 0.012), having a headache (PR 1.67, p = 0.004) and feeling irritable or angry (OR 2.20, p = <0.0001) (Table 1).

**Table 1:** factors associated with feeling concerned about one´s mental health in the face of the COVID-19 pandemic

Variables	cOR (95%CI)	p-value	aOR (95%CI)	p-value
**I am concerned that I may contract COVID-19 during my work (N=729)**				
Strongly agree	Ref	--	Ref	--
Agree	0.76 (0.53; 1.09)	0.137	0.81 (0.49; 1.35)	0.418
Neutral	0.34 (0.20 (0.59)	<0.0001	0.54 (0.31; 0.94)	0.029
Disagree	1		1	
Strongly disagree	0.46 (0.06; 0.83)	0.009	0.44 (0.26; 0.74)	0.002
Not applicable	0.51 (0.29; 0.91)	0.098	0.48 (0.27; 0.88)	0.017
**My supervisors understand my concerns (N=723)**				
Strongly agree	Ref	--	Ref	--
Agree	1.29 (0.85; 1.96)	0.232	1.70 (0.75; 1.83)	0.495
Neutral	0.99 (0.61; 1.59)	0.954	0.92 (0.55; 1.56)	0.750
Disagree	2.73 (1.55; 4.82)	0.001	2.25 (1.22; 4.14)	0.009
Strongly disagree	2.38 (1.28; 4.41)	0.006	2.16 (1.11; 4.21)	0.023
Not applicable	0.40 (0.19; 0.85)	0.016	0.33 (0.15; 0.73)	0.006
**I have received information on methods of self-care (N=723)**				
Strongly agree	Ref	--	Ref	--
Agree	1.27 (0.90; 1.79)	0.182	1.26 (0.86; 1.85)	0.232
Neutral	1.20 (0.66; 2.17)	0.559	1.48 (0.78; 2.81)	0.236
Disagree	2.47 (1.46; 4.18)	0.001	2.44 (1.35; 4.39)	0.003
Strongly disagree	2.08 (1.15; 3.77)	0.015	1.68 (0.88; 3.19)	0.116
Not applicable	0.38 (0.04; 3.74)	0.410	1.34 (0.12; 12.25)	0.811
**Profession (N=723)** Mental health professional	Ref	--	Ref	--
Clinician	0.49 (0.30; 0.79)	0.003	0.41 (0.24; 0.68)	0.001
Public health professional	0.49 (0.21; 1.14)	0.099	0.44 (0.18; 1.08)	0.073
Social worker	1.29 (0.79; 2.08)	0.307	1.08 (0.65; 1.81)	0.762
Other	0.71 (0.40; 1.29)	0.261	0.63 (0.34; 1.17)	0.147
**Factors associated with female gender (Reference = Male respondents)**				
**Symptoms (N=719)**				
Sad	1.85 (1.30; 2.63)	0.001		
Scared	1.49 (1.09; 2.04)	0.012		
Headache	1.67 (1.18; 2.36)	0.004		
Irritable/Angry	2.20 (1.50; 3.23)	<0.0001		
**Recommendations (N=719)**				
Access to wellbeing officer / counsellor	1.46 (1.09; 1.97)	0.012		
Occupational safety	0.61 (0.41; 0.90)	0.014		

### Predictors of mental health concerns

Less than half of the respondents were comfortable and “not overly concerned” about their contracting the virus and were less likely to acknowledge any mental health concerns (aOR 0.044, p = 0.002). The important role of the supervisors was clearly established as participants who disagreed (aOR 2.25, p = 0.009) or strongly disagreed (aOR 2.16, p = 0.023) that the “supervisors understand” their mental health concerns take steps to address them. Consistently across age, and professional groups, the proportion in the category that felt that the supervisors were unconcerned was twice as likely to report anxiety about their mental health. Participants who had not received information on methods of self-care during the COVID-19 response were significantly more likely to report anxiety (aOR 2.44, p = 0.003). Prior experience at managing a health emergency did not influence the feeling of concern. Compared with 48% who did not have previous experience, 59% of those who had previously participated in a health crisis emergency expressed anxiety about their mental health.

### Stigma

Out of the 723 respondents, 44% expressed concerns about being stigmatized and excluded in their own community for serving those affected by COVID-19. Almost half of all social workers (49%) and clinicians (46%) were concerned about being stigmatized, compared to only a third of public health professionals (34%) and mental health professionals (34%).

### Education, training, and MHPSS capacity in COVID-19 response

Despite that 85% of the respondents acknowledged receiving skills and education on self-protect from infection, 70% still expressed concerns about likelihood of contracting COVID-19 during their work. Clinicians (69%) and social workers (70%) were less likely to agree to having received information on self-care than mental health professionals (78%), others (78%) and public health professionals (83%).

Mental health professionals that acknowledged having skills for providing Psychological First Aid (PFA) to both co-workers and those affected by COVID-19 were 63% compared with social workers (41%), public health professionals (17%), or clinicians (33%). However only 26% of the frontline workers recommended training on Psychological First Aid to first responders. About two thirds of the respondents do not know to whom cases of advanced level psychosocial support should be referred.

Majority (70%) of the Frontline workers expressed a strong concern about contracting COVID-19 during their work and 35% wanted additional occupational safety measures and access to personal protective equipment. Only 28% showed interest in relaxation and stress management training and a quarter (26%) preferred flexible schedules for workers that are directly impacted or have a family member that is impacted by a stressful event. The findings were consistent irrespective of professional groups or age groups. However, while more women recommend access to a wellbeing officer and counsellor (OR 1.46, p = 0.012) men emphasised the importance of occupational safety (OR 0.61, p = 0.014).

## Discussion

While more than half of the respondents were from South Sudan (33%), Kenya (13%) and Uganda (12%), the findings corroborate similar evidence collected through community feedback mechanisms within the Risk Communication and Community Engagement technical working group; as well as by protection partners and human rights advocates across the Eastern and Southern Africa Region [3]. With two thirds of respondents (68%) feeling overwhelmed, stressed or anxious, and 33% expressing fear, it is clear that the COVID-19 pandemic has an impact on the mental wellbeing of frontline workers. Consistent with similar research from Italy [4], women were more likely than men to report feelings of sadness, fear, and anger. One potential explanation for this gendered difference could be related to additional burdens associated with gender roles, as women might experience additional stress because of additional demands to care for children who stay at home due to school closures.

**Figure 1:**
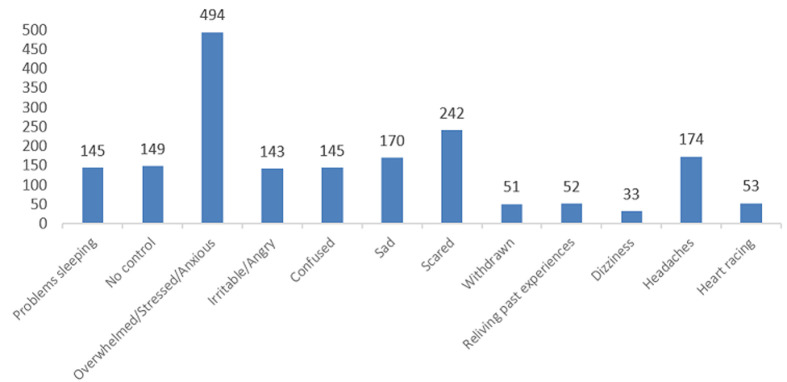
feelings experienced during COVID-19 (by number of respondents)

It is not surprising that respondents who did not feel supported by their supervisors were more than twice as likely to report feeling concerned about their mental health. Another study [5] indicates that poor or lack of social support from supervisors at work increase the risk for mental disorders that are treated with antidepressant medication. Supervisors should support frontline workers to mitigate the risk and allay their concerns about mental health. Such support will prevent exacerbation before it results in burnouts and mental health disorders among frontline workers.

Although frontline workers are celebrated worldwide for their intervention efforts during the ongoing pandemic, there are reports of their exposure to stigma, discrimination and even violence [6]. The comparatively higher concern about stigma by the clinicians than the other health professionals could be attributed to the perceived higher risk of exposure that the clinician group feels which is absent in the latter. The clinicians are more likely to be in contact with positive cases than the other health professionals. The latter group are also more likely to be in greater contact with the community members and have the opportunity to apply their social non-clinical skills than the former group. Further research at community level will improve present understanding about the dynamics of social interaction of the professional categories and its influence on perception of stigma.

Training and skill acquisition do not seem to allay anxiety about mental health. The anxiety is due to the fear of exposing loved ones to the infection. Concerns about becoming infected and infecting others as a result of their work cannot simply be mitigated by sensitizing frontline workers on infection prevention procedures and protocols. Education of the frontline worker is not sufficient to remove the anxiety, but necessary to provide supplies that assure of their protection by reducing the risk of infection. In addition, it may be important to extend occasional counselling and training to the family members. At the same time, it is important to conduct participatory assessments at country-level to better understand the factors contributing to the wellbeing concerns among frontline workers.

Lastly, the large proportion of social workers and clinicians did not receive information on self-care is a potential cause for concern, and their anxiety about wellbeing is well-placed. Considering that clinicians are the first line of medical response in terms of triage, treatment and management of COVID-19 patients, they are more likely to be exposed to the virus. Similarly, social workers might interact with people and communities affected by the coronavirus, which puts them at risk of infection and justifiable may cause some stress. The lack of personal protective equipment, long working hours and the curtailed physical interaction with their families may exacerbate the anxiety feeling. In order to mitigate the risks of frontline workers experiencing severe stress, burnout, or mental health disorders, there should be continuous efforts by national authorities to disseminate information on methods of self-care and stress management to frontline workers across all sectors of the COVID-19 response.

We recommend replicating this and other similar social studies to better understand the extent, cause and nature of mental health concerns that are associated with first responder to health crisis. Supervisors and managers should be trained and sensitized on the importance of mental health, signs of stress and anxiety; and how to support staff members who are experiencing stress and mental health problems. Clearly defined referral pathways and guidelines are necessary and should complement the intervention activities. The provision of basic information on self-care and stress management to communities should be integrated into primary care, and Psychological First Aid skills included in primary health worker education. Practitioners should strengthen their engagement with mental health experts to identify avenues to strengthen frontline workers, managers and supervisors´ capacity to provide basic mental health and psychosocial support, in all sectors of the COVID-19 response. Further research is required to better understand the nature of stigma the various professional categories experience and public perceptions of risk of infection. Ultimately, it was recommended to pursue advocacy towards governments to integrate mental health and psychosocial response in all public health response measures to COVID-19 and mental health systems at large to leverage the efforts made over the last years.

## Conclusion

Further studies will improve current understanding of the mental health and psychological concerns other categories of professional caregivers experienced while responding to the pandemic.

### What is known about this topic

- There is still limited research on the understanding of the mental health and wellbeing of frontline workers that were at the forefront of the preparedness and response to COVID-19.

### What this study adds

- This research is the outcome of a mental health assessment survey of frontline workers in Eastern and Southern Africa regarding COVID-19 pandemic in the region which conducted at the onset of the pandemic;
- It brings crucial information on their capacities and needs and will be used as a baseline for the evolution of frontline workers´ mental health and wellbeing and inform preparedness and response measures in the future.
